# Fast Track Management of Primary Thyroid Lymphoma in the Very Elderly Patient

**DOI:** 10.3390/curroncol30060435

**Published:** 2023-06-15

**Authors:** Pierre Yves Marcy, Frederic Bauduer, Juliette Thariat, Olivier Gisserot, Edouard Ghanassia, Bruno Chetaille, Laurys Boudin, Jean Baptiste Morvan

**Affiliations:** 1 Radiodiagnostics and Interventional Radiology Department, Polyclinics ELSAN Medipole Sud, Quartier Quiez, 83189 Ollioules, France; 2 Department of Hematology, Centre Hospitalier de la côte Basque, College of Health Sciences, Bordeaux University, 64100 Bayonne, France; bauduer.frederic@neuf.fr; 3 Department of Radiation Therapy, Francois Baclesse Cancer Research Institute, Laboratoire de Physique Corpusculaire IN2P3/ENSI CAEN/CNRS UMR 6534, Normandy University, 3 Avenue General Harris, 14076 Caen, France; jthariat@hotmail.com; 4 Department of Medical Oncology, CH Sainte Musse, 54 Rue Sainte-Claire Deville, 83100 Toulon, France; oliviergisserot@gmail.com; 5 Department of Endocrinology, PolyClinics Sainte Therese, 06 quai du mas Coulet, 33200 Sete, France; drghanassia@gmail.com; 6 MEDIPATH Toulon, Pathology Center, 146 Avenue Foch, 83300 Toulon, France; b.chetaille@medipath.fr; 7 Department of Medical Oncology, University Military Hospital Sainte Anne, 2 Boulevard Sainte Anne, BP 600, 83300 Toulon, France; 8 Department of Head & Neck Surgery, University Military Hospital Sainte Anne, 2 Boulevard Sainte Anne, BP 600, 83000 Toulon, France; jbmorvan@hotmail.com

**Keywords:** neck mass, thyroid malignancy, primary thyroid lymphoma, anaplastic thyroid carcinoma, metastasis to thyroid, ultrasonography, FDG PET- CT, MRI, core needle biopsy, prognostic nomogram

## Abstract

A rapid growing cervical mass mobile while swallowing is the most common clinical presentation of severe thyroid malignancy. A 91-year-old female patient with a history of Hashimoto thyroiditis presented with clinical compressive neck symptoms. The patient had gastric Maltoma diagnosed that was surgically resected thirty years ago. A straightforward process was needed to reach full histological diagnosis and initiate prompt therapy. Ultrasound (US) showed a 67 mm hypoechoic left thyroid mass with reticulated pattern without signs of locoregional invasion. Percutaneous trans isthmic US-guided 18G core needle biopsy (CNB) disclosed diffuse large B cell lymphoma of the thyroid gland. FDG PET revealed two distinct thyroid and gastric foci (both SUVmax 39.1). Therapy was initiated rapidly to decrease clinical symptoms in this aggressive stage III primitive malignant thyroid lymphoma. The prognostic nomogram was calculated by using a seven-item scale, which disclosed a one-year overall survival rate of 52%. The patient underwent three R-CVP chemotherapy courses, then refused further treatment and died within five months. Real-time US-guided CNB approach led to rapid patient’s management that was tailored to patient’s characteristics. Transformation of Maltoma into diffuse large B cell lymphoma (DLBCL) into two body areas is deemed to be extremely rare.

## 1. Introduction

A rapid growing painful mass in the neck in the elderly with clinical compression raises three main diagnoses of thyroid malignancy: primary thyroid lymphoma (PTL), anaplastic thyroid carcinoma (ATC) and, less frequently, metastasis to thyroid gland (MTT). PTL is an uncommon disease, representing less than 5% of thyroid malignancies [1]. It was shown that 89% of thyroid lymphomas arise in a context of previous auto-immune disease [2]. PTL includes two main types that have different prognosis and treatment; the aggressive life- threatening diffuse large B cell type (DLBC), particularly in the elderly patient, and the indolent mucosa-associated lymphoid tissue (MALT) lymphoma; mixed subtypes were also reported [3]. Thyroid high frequency ultrasound (US) and fine needle aspiration cytology (FNAC) using immunohistochemistry remain the main modalities used to suggest malignant lymphoma. Percutaneous ultrasound-guided core needle biopsy (CNB) may achieve full accurate diagnosis, thus further limiting the role of invasive thyroid surgery [4]. DLBCL is aggressive, and survival outcome increases with multimodality therapy incorporating monoclonal antibodies, chemotherapy and radiation therapy. Prognosis may vary owing to the heterogeneous nature of thyroid malignant lymphomas [1,3,5,6,7]. Regarding ATC, prognosis is even poorer, differential diagnosis from PTL is urgent and now can be performed by using CNB droplet digital polymerase chain reaction; thus, targeted therapies may greatly improve disease-specific survival of ATC patients [8].

The aim of this paper was to report a life-threatening DLBCL compressive case in a very elderly female patient, to stress the single novel key role of US-guided percutaneous CNB in promptly establishing the histological subtype diagnosis, of FDG PET-CT in the patient’s staging and aggressiveness of disease and, eventually, to discuss prognosis factors in such patients.

## 2. Case Presentation

**Figure 1:**
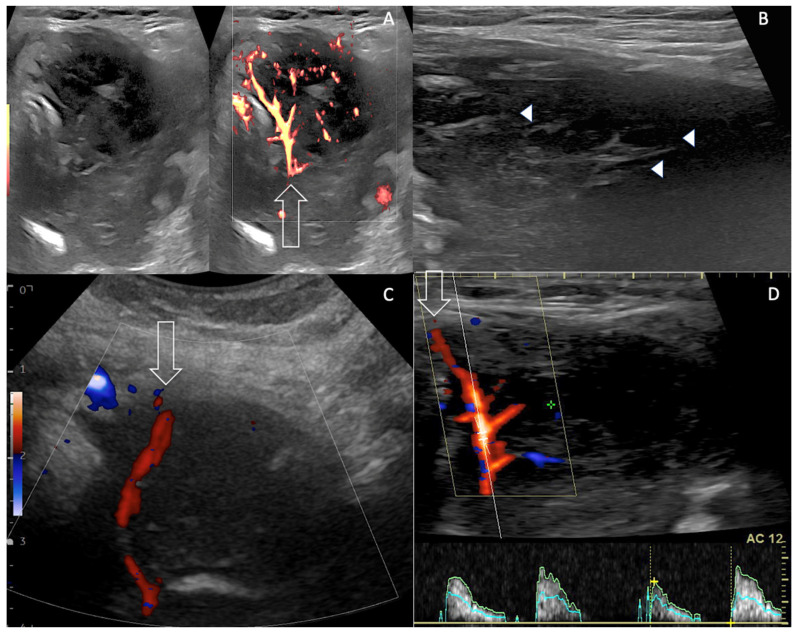
Multiparameter Doppler US assessment of left thyroid mass: reticulated pattern and malignant vasculature. (**A**,**B**). Axial US grey scale and Power Doppler showing EuTIRADS5 67 mm left thyroid mass with intervening echogenic septa-like structures (arrow heads) that correlate with fibrosis on histopathology [9], increased vascularity (arrow), unclear boundaries. “Sword-like” vessel is abnormal aberrant tumour artery merely noticed in dedifferentiated thyroid cancers, ATC and PTL (arrow **A**,**C**,**D**). High resistive index (RI = 1) is shown on spectral analysis (**D**). High RI > 0.75 is typical of malignant neoangiogenesis [13].

A 91-year-old female patient presented with rapidly progressive dysphonia and intense painful dysphagia. The patient did not demonstrate any cognitive deterioration. The patient’s body mass index (BMI) was 28.5 kg/m^2^, despite a recent weight loss of 3 kg. The patient did not experience a recent fever or sweats. The worsening of eastern cooperative oncology group performance status (ECOG-PS) scoring from grade zero to grade 2 was concomitant to the progression of the recent clinical symptoms [9]. Physical examination showed a firm and somewhat painful mass in the left anterior part of the neck, which moved while swallowing without inflammatory local sign. Neither peripheral lymphadenopathy nor hepatosplenomegaly was detected. The patient did not complain of any abdominal pain. Her past-medical history included a stage-IE gastric MALT lymphoma, which was diagnosed and treated by partial gastrectomy thirty years ago [10]. The patient was shortly lost to follow-up; she did not have any history of radiation exposure, family history of thyroid-related disorders or familial malignancy. Her only medication was Levothyroxine 75 for 30 years, due to Hashimoto’s thyroiditis (HT)-related hypothyroidism. A US examination of the neck disclosed a 67 mm firm strongly hypoechoic neck mass originating from the left thyroid that strongly displaced the cervical trachea. This vascularized EuTIRADS 5 mass [11] displayed typical reticulated pattern [12] and a “sword sign” neovasculature [Figure 1] [13]. US did not disclose neither jugular vein invasion/thrombosis nor local lymphadenopathy in the neck in the II-VI levels, according to Robbins ’s classification [14].

**Figure 2:**
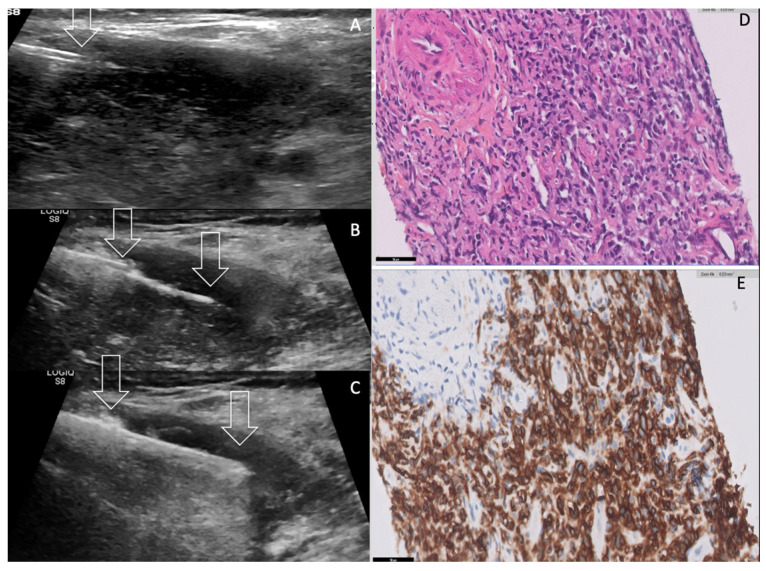
Core Needle Biopsy (CNB) technique and results. (**A**) CNB was performed using horizontal percutaneous right-to-left trans isthmic 17G needle trocar (**A**–**C**). Note advance of trocar tip within the left thyroid mass (arrow). (**B**) The semi automatic 18G CNB stylet of the cutting needle (between arrows) was then advanced into the tumour tissue, away from the thyroid capsule. (**C**) Firing the cutting needle (between arrows) was performed after colour Doppler assessment (not shown) to avoid arterial damage. (**D**,**E**) Biopsy specimen histopathology showing 15 mm long monomorphic sheets of large atypical lymphoid cells, containing numerous easily identifiable mitoses (Haematoxylin and Eosin staining × 400). Immunostaining with the pan B anti-CD20 antibody showed strong positivity (in brown) of all the atypical cells; ×400. CD20+ and CD3- were the signature of B lymphoma. Note the absence of lymphocytic thyroiditis and germinal centres. See also Video File S1.

A CT-scan assessment showed a well-defined left homogeneous lobar thyroid mass, which displaced the trachea anteriorly and laterally, the oesophagus posteriorly, without macroscopical invasion of trachea, oesophagus or internal jugular vein. Contrast enhanced body CT ruled out hepatosplenomegaly, any superficial and deep lymph node of neck thoracic abdominal and pelvic location. Subtle thickening without nodule or mass of the operated stomach remnant was noted. A complementary MRI examination showed the compressive left thyroid mass displaying homogeneous hyposignal T1 hypersignal T2 and dramatically low apparent diffusion coefficient (ADC). At initial work-up, biology tests showed normality of the patient’s blood count basic metabolite profile and liver function; C Reactive Protein (CRP) was moderately elevated 12.7 (Nl < 5.0). Serum Lactico dehydrogenase (LDH) was 270 U/L (Nl < 250) and beta 2 microglobulin= 4.1 mg/L (normal <2.0); Albuminemia = 32.2 g/L (40.4–47.6); Creatinin Clearance = 45 mL/mn/1.73 m^2^. The TSH serum level was normal = 1.5 IU/mL (Levothyroxine 75 medication). Positive serum level of anti-thyroperoxidase antibodies (ATPOAb) were in favour of pre-existing HT. As the patient’s dysphagia and dyspnoea rapidly worsened, a percutaneous FNAC and trans-isthmic real-time ultrasound-guided core needle biopsy (CNB) of the thyroid mass were performed in semi-upright position under local anaesthesia [Figure 2, Video File S1].

FNAC smears were cellular and comprised of monomorphic population of large atypical lymphoid cells with indented nuclei. FNAC specimen immunostaining analysis was positive for CD20, CD10, BCL6 and negative for CD3, CD30, BCL2 and also for EBV. Ki 67 index was 80%. FNAC smears were consistent with aggressive B cell-PTL. Complementary histopathological examination of the six biopsy specimens showed diffuse sheets of large transformed lymphoid cells of centroblastic and immunoblastic appearance that were suggestive of a high- grade lymphoma. Complementary immunochemistry demonstrated these cells to express the pan B cell marker CD20 along with the expression of the centro-germinative markers CD10 and BCL6. These cells did not express the T cell markers CD3 and CD5, nor the post-germinative marker MUM1. Finally, the diagnosis of a diffuse large B cell lymphoma, not otherwise specified, of Germinal Centre (GC) type was retained. Histopathology also highlighted the presence of remnants of thyroid epithelium trapped into the malignant lymphomatous proliferation. However, no associated low-grade lymphoma could be seen on all six biopsy specimens. Immediate and 1 h post CNB US assessment did not highlight any haemorrhagic complication.

**Figure 3:**
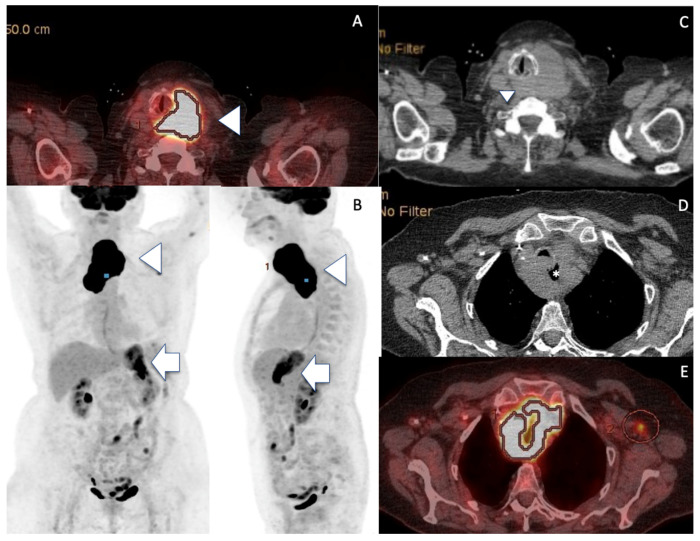
FDG PET-CT showing malignant thyroid and gastric uptake. (**A**,**B**) FDG-PET discloses uptake foci into the enlarged left thyroid (arrowheads) but also into the stomach (arrows, SUV at 39.1 for both sites). (**C**–**E**) Note the left PTL mass (arrowhead) spreading behind the larynx/trachea, encasing upper oesophagus (*).

FDG PET-CT disclosed high uptake foci into both left thyroid mass and gastric remnant, and no medullary bone involvement [Figure 3]. The patient underwent three courses of R-CVP (rituximab, cyclophosphamide, vincristine and prednisolone). A partial clinical therapeutic response was obtained following this regimen, partial regression of the neck compression symptoms was noted, the tolerance was quite satisfactory. However, the patient refused to undergo the fourth course and the involved field radiotherapy. The patient’s compressive symptoms worsened soon after three courses of chemotherapy and did not improve, despite prednisolone 0.5 mg/kg oral medication. The patient died five months after the initial work-up, following a rapid deterioration of her general condition.

## 3. Discussion

Clinically, a thyroid mass mobility while swallowing is the main symptom proving the thyroid origin; however, misdiagnosis may occur when an extra capsular aggressive thyroid tumour infiltrates the adjacent neck muscle and hinders thyroid movement when swallowing. A painful mass of the neck associated with compressive symptoms in the elderly is very suggestive of aggressive thyroid malignancy. This includes primary thyroid lymphoma (PTL) [15] anaplastic thyroid carcinoma (ATC) [16] and metastasis to the thyroid gland (MTT) [17], [Table 1]. PTL is a rare type of malignancy accounting for 1 to 5% of thyroid neoplasms and 1 to 7% of extranodal lymphomas. It is associated with a female predominance (2.5/1 ratio), a higher incidence after the sixth decade and more aggressive types in very elderly patients [18]. The other second aggressive thyroid tumour is ATC, which is also considered to be a rare and highly aggressive malignant tumour, accounting for two to three percent of all thyroid gland neoplasms. ATC continues to be one of the most deadly diseases worldwide and carries a very poor prognosis, with an average survival of five months [16,19]. As disease-specific survival may be significantly improved by using targeted therapies, differentiating ATC from PTL is of utmost importance and becomes an emergency. Given the aggressive course and resistance to chemotherapy, radiotherapy and radioactive iodine, and the potential to identify a targetable mutation, all patients must undergo expeditious staging, histological confirmation and broad-spectrum next-generation sequencing testing on CNB specimens [20].

The third diagnosis, MTT is rather uncommon clinically (0.24%), compared to autopsy series (1.9–24%); the reported incidence ranges from 0.36% to 3% in all thyroid malignancies [17,21,22,23,24]. It may present as a synchronous or metachronous solitary thyroid mass in 71% of the cases. When the neck mass occurs before discovery of the primary tumour, diagnosis can be a challenging task. Although the overall prognosis is poor, a subset of patients with oligometastasis can be managed surgically with a reported average survival of 43.2 months after thyroid surgery. Primary tumours mostly include renal colorectal lung breast cancers and, more rarely, sarcomas and melanomas. Prognosis is generally poor, but it depends on the characteristics of the primary tumour [24].

Regarding imaging in the late nineties before the era of high frequency ultrasound, the sonographic finding of PTL was misinterpreted as “pseudocystic mass” by using 7 MHz US probe [25]. Now, by using 13–17 MHz US probe, the regular architectural layout of lymphoma tissue typically displays solid strongly hypoechoic pattern corresponding to EuTIRADS5 mass, combined with a reticulated aspect frequently intermingled with coexistent thyroiditis. The relative risk of a HT patient to develop PTL was estimated to be 40 to 80 times greater than in the general population and to take on average 20 to 30 years to develop after the onset of lymphocytic thyroiditis [2]. The inflammatory fibrosis displays echogenic fibrous strands and creates outline lobulation; reactive lymph nodes according to Robbins classification can be found in the central compartment, the so-called level VI [14]. Identifying such HT features on the right thyroid lobe in the present case suggested the proper diagnosis, thus leading to left mass CNB planning. The early transformation of HT into PTL is difficult to assess, and sometimes relies on the careful and subtle detection of a hypoechoic mass of convex contours within the underlying hypoechoic LT tissue. Unclear boundaries internal jugular vein invasion/thrombosis and lymphadenopathies are less frequent than in ATC [26,27]. Identifying echogenic fibrous strands [Figure 1B] that are typical features in favour of PTL could be the key- point for differentiating PTL from ATC. However, it is very difficult to differentiate PTL from ATC by using ultrasound alone in most of the cases. Takashima et al. reported three typical PTL features on CT imaging, including a solitary nodule (80%), multiple nodules (13%) or a diffuse goitre (7%). Tumour involvement of both lobes of the thyroid gland was noted in 47% [28]. In this series, all patients had coexistent HT, and 87% complained of a rapid enlarging thyroid mass, as in the present case report. A comparison to ATC showed that both ATC and PTL have a strong tendency to compress the surrounding neck structures, and PTL is less prone to infiltrate than ATC. Two typical CT features may help to differentiate PTL from ATC in terms of imaging: necrosis calcification and tumour composition. Distinctively, ATC tissue displays heterogeneous attenuation due to necrotic areas and also heterogeneous rim-like metastatic lymph nodes. ATC gross calcifications (mean size: 8.2 mm) are usually multiple in number and are presumed to be a delayed marker of tumour necrosis. Conversely, PTL tissue typically displays homogeneous attenuation and homogeneous lymphadenopathies, but neither calcification nor necrosis [29]. Concerning the final diagnosis of MTT, the accuracy of FNAC is lower than that expected because of pre-existence of non-metastatic thyroid nodules in 44.2%. Though FNAC immunohistochemical (IHC) analysis is very useful, this requires information about the primary tumor, so that the proper antibodies can be used for IHC analysis; therefore, FNAC accuracy rate was 73.7–87%. False negative results include oesophagus, cervix, renal clear cell carcinoma RCC and melanoma [17,21,22,23,24]; thus, CNB should be also selected in appropriate cases of MTT.

In the present case, histological diagnosis from a biopsy specimen was needed urgently to distinguish PTL from ATC and MTT [Table 1]. As the literature reported a better diagnostic accuracy for CNB (100%), compared to 30–90% for FNAC, we performed both procedures concomitantly [30,31,32,33]. This was performed by using percutaneous ultrasound-guidance rather than a more risky and invasive classical surgical biopsy procedure. As a matter of fact, the percutaneous real-time ultrasound-guided 18G-CNB has many advantages over open surgical thyroid biopsy. The percutaneous biopsy procedure can be promptly planned in the interventional radiology department suite, monitored on real time US guidance and performed in a sitting position. This was particularly useful in this very elderly fragile patient. Percutaneous CNB remains a minimally invasive percutaneous procedure with no blade incision and one single needle tract, allowing multiple CNB sheets thanks to the use of trocar guide, thus minimizing the risk of bleeding and tumour track seeding. Using the trans-isthmic route enables to stabilize the CNB needle in a very safe and most easily recognizable in-plane US monitoring of the echoic CNB needle long axis to the target. The bleeding risk of large core needle neck biopsy is also dramatically reduced, as colour Doppler US displays all tumour vessels along the needle tract to prevent accidental core-needle puncturing [30]. Moreover, using semi-automatic mode, the CNB needle permits a very useful and safe step-by-step biopsy procedure under colour Doppler US monitoring: removing the stylet, inserting the biopsy gun through the introducer, pushing the stylet into the lesion manually while avoiding any arterial tumoral vessel, then firing the cutting canula away from the thyroid capsule and carotid artery and removing tissue from the lesion [Figure 2A–C] [Video S1, Figure S1].

A recent meta analysis comparing CNB to FNA showed that thyroid CNB has a superior diagnostic value, with a sensitivity and positive predictive value PPV of 94.3% and 100% for PTL versus 80.1% and 100% for ATC, respectively [32]. CNB also reduces the need for diagnostic surgery to 6.2% for PTL patients. CNB is safe, well-tolerated and associated with a low incidence of complications when the procedure is performed by an experienced operator; thus, this should be proposed as the first diagnostic procedure in case of suspicion of PTL [31,33,34].

**Table 1:** Comparison of imaging features and prognosis in PTL, ATC, MTT patients presenting with growing neck mass.

**Etiologies** % of malignancies Sex Ratio F/M	**PTL** (1–5%) 2.5/1	**ATC** (2–3%) 1.5–2/1	**MTT** (0.36%, 1.9–24% autopsy) 1.1–1.4/1
**Hashimoto’s** **Thyroiditis**	Common	Uncommon	Uncommon
**Thyroid Function**	Hypothyroidism (concomitant HT)	Euthyroidism Hyperthyroidism	Euthyroidism Hypo/hyperthyroidism Hypoparathyroidism *
**Diagnosis** FNA sensitivity CNB sensitivity	44.8% 94.3%	54% 80.1%	73.7–87% Useful in inconclusive cases
**Imaging Features** **US Doppler** EuTIRADS score **IJV invasion** **Computed** **Tomography**	Reticular pattern More homogeneous than thyroid carcinoma No necrosis/calcification Sword sign Eu-TIRADS 5 Rare No cystic necrosis No gross calcification Homogeneous Nodes +/0 Mild vasculature Unclear boundaries + Vessel invasion not usual Tracheal compression +	No reticular pattern Heterogeneous Necrosis /calcification Sword sign Eu-TIRADS 5 Not rare Cystic necrosis Gross calcifications Heterogeneous Nodes ++/− Few vessels Unclear boundaries ++ Vessel invasion (33%) Tracheal compression ++	No reticular pattern Inhomogeneous Depends on pre-existing thyroid No Sword sign Eu-TIRADS 5 > Eu-TIRADS 4 Exceptional No cystic necrosis No gross calcification Nodular Nodes +/− Increased Vasculature (renal) Unclear boundaries 0/+ Vessel invasion very rare (renal) Tracheal compression 0/+
**PET CT** **median SUVmax**	+++ 22.7	+++ 24.8	++/- variable
**One year OS** **5 years OS** **Average survival**	84.66% (52%) 71.66% (24%) Depends on subtype, stage, age	20% - 5months	- 58% 43.2mo (after surgery) Depends on primary histology

HT patients demonstrated a (x40–80) increased risk for developing PTL [2,35], especially Maltoma, whereas 78% of PTL patients had some evidence of HT subtype [1,3,4,5,12,15,32,35]. Dysthyroidism is common in long standing HT, and was exceptionally reported in cases of ATC ([36] or MTT [36,37] and in iatrogenic cases [38]. *Hypoparathyroidism was recently reported as due to concomitant thyroid and parathyroid gland metastasis [37]. CNB is a rapid and safe procedure with higher performance compared to FNAC in identifying the histotype CNB is a rapid and safe procedure with higher performance compared to FNAC in identifying the histotype of large and rapidly growing thyroid masses [35,39,40]. The identification of clonal Ig gene rearrangements allows a differential diagnosis between PTL and HT [39,40]. Homogeneous vascularized mass without calcification and cystic necrosis but with posterior acoustic enhancement are typical features of PTL compared to ATC [12,25,29] and MTT [13,14,17,18,19,20]. The invasion of the internal jugular vein (IJV) occurs exceptionally in PTL [26] and MTT [17,18,19,20,21,22,23,24], and is quite common in ATC (33%) [28]. Vasculature was reported to be increased in PTL [Figure 1] when compared to ATC. MTT can display hypervasculature in renal clear cell carcinoma (RCC)-related MTT as well as arterialized intrajugular vein tumour thrombus [27,29,41,42]. Overall survival (OS) was found to be 52% and 24% at one year and five years in thyroid DLBCL versus 84.66% and 71.66% in all PTL, respectively [43,44,45]. MTT OS improves when surgical resection can be performed [21].

Sharma et al. reported an extensive review of 47 PTL cases, mean age 67 years old, who presented with a neck mass in 89.4%, dysphagia in 46.8%, hoarseness in 40.4% and breathing difficulty in 27.7%, neck pain in 10%, weight loss in 14.9% and B symptoms in 10.6% [30]. A rapidly enlarging mass was reported in 29% of DLBCL (67.5 years) versus 7% of Maltomas (63.2 years). Compression symptoms were far more frequent in DLBCL compared to Maltomas, as follows: dysphagia 27% vs. 13%, cough 15% vs. 7%, dyspnoea 17% vs. 3%, hoarseness: 4.9% vs. 0%, respectively [34].

One can observe DLBCL occurring de novo or following the transformation of a MALT type. Apart from the thyroid gland, MALT lymphomas are also described in the stomach, salivary glands, lung, intestine, breast, ocular adnexa and skin [45]. Whole-body PET FDG CT-scan highlights the precise location of lymphoma uptake(s); thus, it is necessary for tumour staging and response assessment [46,47]. Aggressive lymphoma FDG PET is highly correlated to high SUVmax values, is sensitive for bone marrow involvement and may obviate need for lymphoma staging standard bone biopsy. Thus, the absence of bone marrow uptake in the present case justifies that bone marrow biopsy was not performed in our present case report.

As an indolent disorder, thyroid MALT lymphoma has a good prognosis compared to DLBCL [3], all Maltomas were staged IE or IIE and alive, whereas almost 10% of DLBCL patients displayed stage III or IV and died with a disease [3]. The transformation of MALT into DLBCL type was reported in less than 10% of the cases [45]. Although histology CNB specimens did not formally prove that the thyroid DLBCL arose from the transformation of a pre-existing low grade MALT lymphoma, the existence of a history of Hashimoto’s thyroiditis with positive ATPO Ab, the presence of contralateral thyroid HT features on US assessment, the previous MALT lymphoma of the stomach thirty years ago and similar very high FDG gastric and thyroid uptake values strongly favour this hypothesis. Noteworthy, clinical signs were mainly related to the thyroid localization, and no gastric complaints nor CT focal nodule or mass of the remnant stomach were noted. Acknowledging the absence of histological documentation (planning gastric endoscopy biopsy was judged unethical in this very elderly patient), we speculated from the very high SUV (SUV was 39.1 on stomach and thyroid sites) on the FDG PET-CT about a synchronous DLBCL occurrence at the initial gastric site, in addition to the histologically proven thyroid high-grade lymphoma. Indeed, a SUVmax value over 13 definitely indicates high-grade lymphoma in the present case [47]. Of note, similar very high FDG PET-CT SUVmax values were also reported in ATC [Table 1] [16].

The multisite occurrence of DLBCL/MALT lymphoma is a rare event, as 6% of PTL patients presented with distant lymphatic tumour, which might spread to the stomach, small bowel or colon [15]. Kushwaha et al. [48] reported a synchronous DLBCL of bilateral submandibular glands and a thyroid Maltoma in a 65-year old woman. Bauduer et al. reported a relapse of MALT lymphoma in the orbit and salivary glands nine years after an initial gastric localization [49]. Hao et al. reported on a primary nasal DLBCL presenting with synchronous pulmonary involvement [50].

Regarding therapy, MALT lymphoma, the so-called “indolent disorder”, needs an accurate initial work-up, appropriate therapy and a prolonged follow-up, given the possibility of relapse and high grade malignancy transformation. A mass of the neck is the most typical clinical presentation reported in 37% versus 22% in DLBCL [3]. Long-term disease free survival was obtained in patients with localized (IE-IIE) MALT lymphoma using either local therapies, i.e., surgery and/or radiotherapy or immunochemotherapy (Rituximab). Nevertheless, therapy relapses are not uncommon [44]. Single chemotherapy or radiotherapy alone was administered for indolent lymphomas, while combination therapy is recommended treatment proved to achieve a better outcome for overall survival and disease-free survival than a single modality therapy. DLBCL or mixed forms (DLBCL/MALT) usually require systemic immunochemotherapy (R-CHOP being the most popular regimen) plus subsequent radiotherapy. In the present case, the treatment was determined on several factors including age tumour staging histological subtypes, clinical symptoms and pre-existing pathology. The combination of systemic chemotherapy and local–regional radiotherapy was eventually the selected foundation of the proposed treatment at multidisciplinary council. Considering the patient’s benefit/risk ratio at this advanced age (91 years), our planned therapeutic strategy included four courses of chemotherapy (R-CVP (Rituximab with Cyclophosphamide, Leurocristine and MethylPrednisolone) instead of six courses of R-miniCHOP (R-CHOP, Rituximab with Cyclophosphamide, Doxorubicin, Leurocristine and Prednisolone) due to the risk of poorer tolerance [51,52]. Radiotherapy was told to the patient to be associated with significant side effects such as acute inflammation of the nearby salivary glands, oropharyngeal and oesophageal mucosae. Following three chemotherapy courses, the patient refused to undergo the fourth R-CVP course and any additional treatment and, thus, died rapidly.

The function of surgery in the present case was ruled out as “medical debulking” (by using steroids and chemotherapy) was the first emergency medical option. Inherent surgical risks include general anaesthesia, difficult tracheal intubation, recurrent laryngeal nerve deficit, hypoparathyroidism and tracheal oesophageal wound injury. Moreover, the more rapid “medical debulking”, the less potential risk of tracheotomy/thyroid isthmus or trachea tumour infiltration.

In addition, a study performed by the Mayo Clinic showed that patients undergoing diagnostic biopsy plus adjuvant chemotherapy alone had a more effective complete remission than those undergoing surgical debulking plus adjuvant therapy [53].

However, we believe that urgent percutaneous biopsy and urgent chemotherapy initiation via a PICC line (also implanted in a sitting position) were the most appropriate option in this very elderly patient presenting with aggressive and compressive clinical symptoms.

Regarding the patient’s prognosis, Zhu et al. reported a very meaningful work with the help of multivariate Cox regression analysis in PTL patients. Those authors indicated that independent predictors of overall survival include age, Ann Arbor stage, histological subtype, surgery, chemotherapy, radiation therapy and marital status. By using a dedicated seven item-scale, the one year overall survival estimate was 52% according to the prognostic nomogram (age: 91 years, DLBCL at histology, Ann Arbor stage III, surgery: none, chemotherapy: partial, radiation: none and marital status: none) [43]. The one-year probability of lymphoma-specific survival estimate was 20%, and it was strongly consistent with the actual patient’s survival of five months.

Additionally, Ha et al. reported that the abdominal cavity and gastrointestinal tract were the most common sites of failure in PTL patients [44]. Those data are of prime importance to plainly inform the patient’s close relatives and speak the truth about the fatal outcome in the very elderly patient presenting with such rare tumours.

## 4. Conclusions and Future Perspectives

This case report led to the following conclusions. Firstly, the management of lymphomas in very old individuals will become more frequent in daily practice and should be tailored to each particular case [54]. Secondly, MALT lymphoma transformation into a high-grade malignancy in the thyroid gland and also in the stomach is extremely rare. Thirdly, patient’s quality of life is a main issue when considering diagnosis and treatment procedures in the very elderly. We were in accordance with this point by conducting fast-track management and quick back-home return. Fourthly, Doppler US allows clear tumour diagnosis and evaluation, enables safe and fast CNB of the neck mass and prompt R-CVP chemotherapy initiation. Fifthly and lastly, the 7- items dedicated prognostic nomogram is particularly useful to accurately predict the individual PTL outcome to relatives.

As histopathological examination is the gold standard for the diagnosis of PTL, US-guided CNB of thyroid mass should be introduced in routine clinical practice by experienced hands. This will likely decrease the burden of repeat FNAC, potentially obviate the need for invasive diagnostic procedures such as open incisional biopsy or thyroid lobectomy and avoid any harmful delay in the very elderly patient’s medical care. The best management for PTL still warrants further study, as most literature analyses were based on limited sample sizes; thus, a large randomized controlled trial is still needed.

## Data Availability

No Data were created.

## Supplementary Materials

The following supporting information can be downloaded at: https://www.mdpi.com/article/10.3390/curroncol30060435/s1, Video S1. Percutaneous US guided-CNB was performed by using in-plane horizontal percutaneous right-to-left trans isthmic 17G needle trocar. CNB trocar is shown in-plane within the left thyroid mass. The semi-automatic 18G CNB stylet of the cutting needle is advanced into the tumour tissue, away from the thyroid capsule. Firing the cutting needle is performed after colour Doppler assessment (not shown) to rule out arterial damage. Retrieval of cutting needle and of biopsy specimen is performed; then, the trocar is slightly angulated and another biopsy is performed [2,29]. Figure S1: CNB procedure.
