# Supplementary figures and images for: Fast Track Management of Primary Thyroid Lymphoma in the Very Elderly Patient
Source: Curr Oncol. 2023 Jun 15;30(6):5816–27. doi: 10.3390/curroncol30060435 (PMC10297318; doi:10.3390/curroncol30060435)

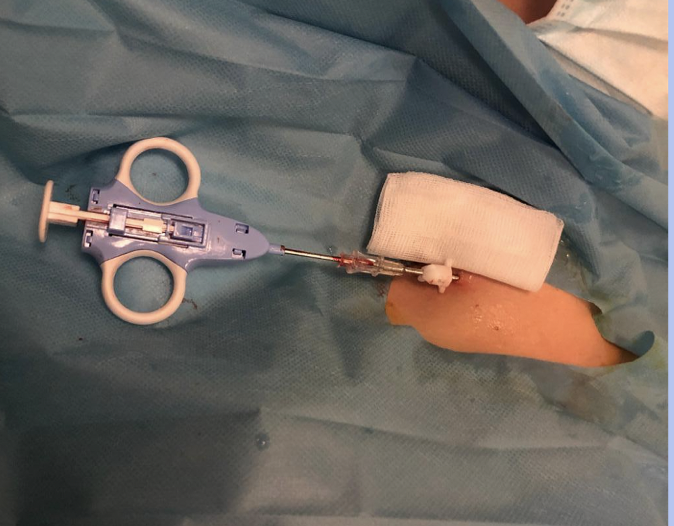

Supplement: Supplementary file 1 [file curroncol-30-00435-s001.zip › curroncol-2425803-Figure S1.png]
